# Insights from mathematical modelling on the proposed WHO 2030 goals for scabies

**DOI:** 10.12688/gatesopenres.13064.1

**Published:** 2019-09-20

**Authors:** Michael Marks, Jodie McVernon, Daniel Engelman, John Kaldor, Andrew Steer

**Affiliations:** 1 London School of Hygiene & Tropical Medicine, London, UK; 2 The Peter Doherty Institute for Infection and Immunity, University of Melbourne, Melbourne, Australia; 3 Murdoch Childrens Research Institute, Melbourne, Australia; 4 Kirby Institute, University of New South Wales, Sydney, Australia

**Keywords:** scabies, modelling, neglected tropical diseases

## Abstract

Scabies was adopted by the World Health Organization (WHO) as a Neglected Tropical Disease in 2017. There is currently no formal global scabies control programmes or existing WHO guidelines on scabies control although at least two countries, Fiji and Ethiopia, have adopted national approaches to scabies control. In February 2019 WHO held a first Informal Consultation on a Framework for Scabies Control, in part as a response to multiple national requests for guidance on public health management in high disease burden areas. Below we outline control strategies proposed at this meeting and summarise the role that modelling can play in supporting the development of evidence to translate these proposals into formal WHO recommendations and national and global control programmes. Provisional proposals discussed at the WHO Informal Consultation for a scabies control programme include the use of mass drug administration when the community prevalence of scabies is ≥ 10% (generally considered to reflect a childhood prevalence of at least 20%) and the use of intensified case management when the prevalence is below 10%.

## Background

Scabies is caused by a microscopic mite ( *Sarcoptes scabiei* var. *hominis*) ( Chosidow, 2006). Female mites burrow into human skin to lay eggs. In classic scabies, the infestation – typically 5 to 15 female mites – and burrowing causes an allergic reaction in the host that triggers severe itching and pain that may interfere with everyday activities, including eating, sleeping, working and studying. Secondary skin lesions then develop, including papules, vesicles and nodules. Rarely, patients may develop crusted (previously known as ‘Norwegian’) scabies, which is characterised by plaques and extensive scales containing millions of mites. This form is highly infectious and associated with a high mortality.

Scabies is transmitted via direct human-to-human contact, making individuals living in overcrowded environments in the poorest of the world’s communities particularly susceptible to developing scabies. Scabies is not zoonotic and cannot be transmitted to humans from dogs or other animals with mange. It is not waterborne and does not appear to be associated with poor hygiene. Skin conditions such as scabies are so common among children in some countries that parents may not consider a skin condition as a primary reason to seek medical treatment for their child.

Primary treatment in individual cases is usually topical and is often effective in resolving scabies in the patient. Of the existing topical agents, permethrin is probably the most effective treatment for scabies. Although topical treatments are efficacious, adherence can be compromised by skin irritation and inconvenience as they need to be applied to the whole body for 8 hours or more. Ivermectin is a highly effective oral treatment, and now approved for second-line use in several countries including France and Australia. There is also widespread experience utilising ivermectin as part of mass drug administration (MDA) for the control of other NTDs.

Several studies now indicate that MDA is an effective strategy for the control of scabies ( Engelman *et al.*, 2018). Studies using both topical and ivermectin-based MDA strategies have demonstrated reductions in scabies prevalence of up to 90%. In the SHIFT randomised trial, conducted in Fiji ( Romani *et al.*, 2015), ivermectin-based MDA reduced the prevalence of scabies by 94% at 12 months. Similar results were seen in the AIM study in the Solomon Islands which enrolled more than 25,000 participants who received ivermectin-based MDA ( Romani *et al.*, 2019a). Follow-up in these study sites at 24 and 36 months respectively demonstrate a sustained reduction in prevalence of both scabies and impetigo following MDA ( Marks *et al.*, 2019; Romani *et al.*, 2019b).

## Mathematical modelling of transmission and MDA

Modelling for scabies is in its infancy. Only a single paper ( Lydeamore *et al.*, 2019) has used a model incorporating mite life stages, and drug actions in relation to these stages, to explore questions related to the control of scabies. Lydeamore *et al.* used a mathematical model of scabies life-cycle and transmission dynamics in a homogeneously mixing population to simulate the impact of mass drug treatment strategies acting on egg and mite life cycle stages (ovicidal) or adult mites alone (non-ovicidal). Ovicidal interventions are currently based on topical treatments. While the model predicts that at high coverage rates the treatment strategies are highly effective, in practice these topical therapies are associated with poor compliance.

**Figure 1.**
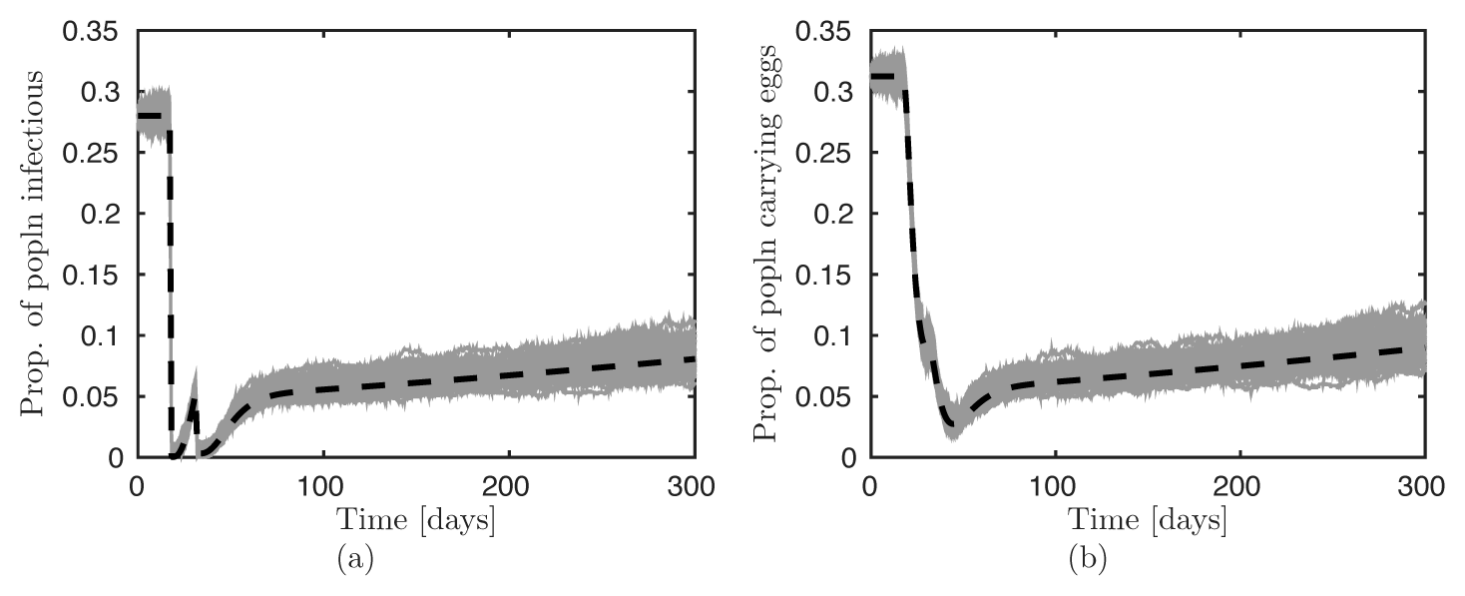
A total of 50 realisations of the Markov model (grey lines), using the Gillespie algorithm, compared with the mean-field approximation (black dotted line) with an MDA program consisting of two optimally timed non-ovicidal treatment doses on ( **a**) the proportion of infected individuals and ( **b**) the proportion of the population with eggs. Figure 1 has been reproduced with permission from Lydeamore MJ, Campbell PT, Regan DG, *et al.* A biological model of scabies infection dynamics and treatment informs mass drug administration strategies to increase the likelihood of elimination. Math Biosci 2019; 309:163–173.

For the non-ovicidal intervention (corresponding to the oral agent ivermectin), the model predicts that at least two optimally-timed doses are required to achieve short term control. However, the model also demonstrates that rebound infection is inevitable following this strategy, even assuming optimal conditions (100% efficacy, 100% coverage, no reimportation), due to residual persistence of eggs. Figure 1a demonstrates this point by showing the proportion of individuals who are infectious (i.e. harbouring pregnant mites) after the first and second doses of therapy. The spike between doses is due to hatching eggs, justifying the need for the second dose. Figure 1b (right) shows the proportion with residual eggs, which does not reach zero even after two doses.

**Figure 2.**
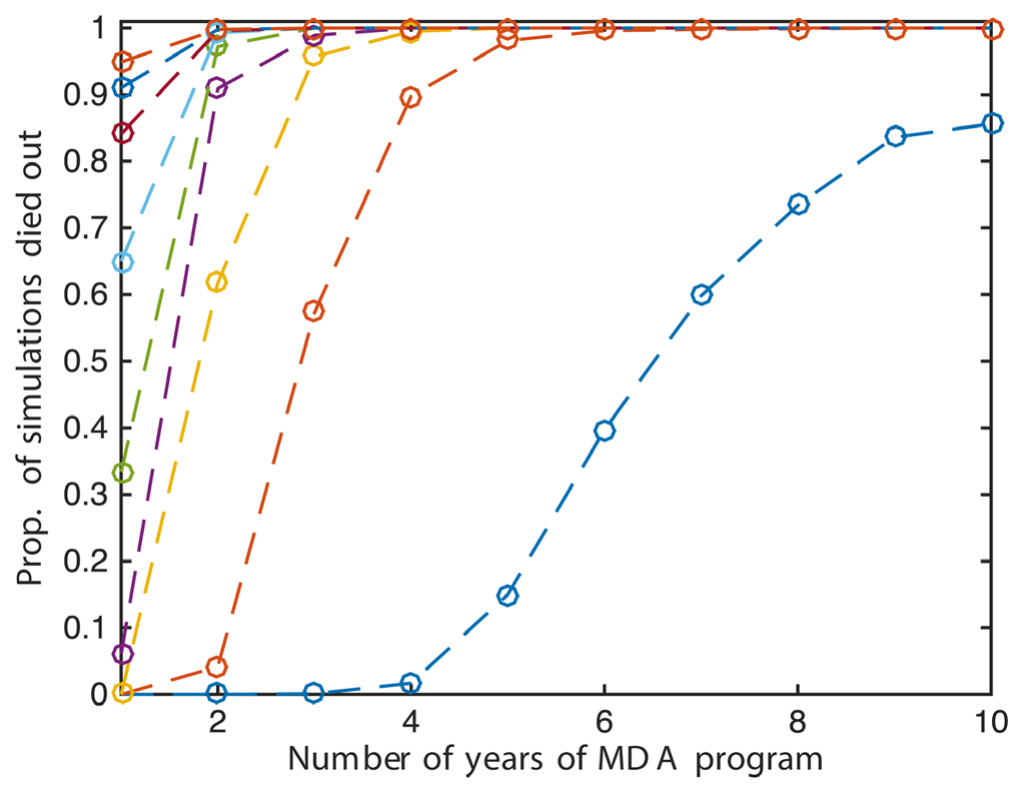
Mean of 1000 simulations of the Markov chain, X(t), which experienced die-out for varying numbers of successive optimally timed non-ovicidal MDA treatments, with the program being repeated annually. Figure 2 has been reproduced with permission from Lydeamore MJ, Campbell PT, Regan DG, *et al.* A biological model of scabies infection dynamics and treatment informs mass drug administration strategies to increase the likelihood of elimination. Math Biosci 2019; 309:163–173.

The model predicts that the likelihood of elimination is enhanced by repeated annual cycles of a multi-dose strategy, which drive down the proportion harbouring eggs over successive treatment courses. In this simplified system, a two dose schedule achieves 50% likelihood of elimination after six years of implementation, and 80% after ten years ( Figure 2). Further exploration in a more realistically configured population model is required to support policy.

## What can modelling tell us about the currently proposed goals

Prior to 2020 there have been no established goals for scabies control but WHO proposes including targets related to scabies in the next Neglected Tropical Disease (NTD) roadmap. Alongside incorporation of scabies into countries’ Universal Health Care plans, it is likely that ivermectin-based MDA will form a substantial component of the control strategy in many highly endemic countries. Modelling may help programmes to determine the optimal threshold for initiating and ceasing, and inform the optimal number and timing of MDA rounds.

## Are there risks that need to be mitigated to achieve and maintain the stated goals?

The absence of any current scabies control programmes represents a major risk to eventual control of scabies. Unlike other NTD programmes for which MDA has been determined to be a key control strategy, there is no drug donation or funding scheme to support implementation of scabies control interventions programmatically. A strengthened evidence base to support WHO recommendations will increase the likelihood of securing political and financial support for scabies control.

As with other NTD programmes, systematic non-participation in MDA may pose a barrier to control, although this has not been formally evaluated within the context of a scabies control programme. Modelling for other NTDs has shown that non-participation can have a substantial effect on impact ( Dyson *et al.*, 2017). Current requirements for two doses of treatment to be delivered during MDA might be anticipated to reduce participation. Future modelling work could determine the effect of systematic non-participation on the outcome of control interventions for scabies whilst ongoing trials will provide insight into whether a single dose MDA regime is sufficiently effective that it may be used in place of a two dose regime.

A small proportion of individuals with scabies may be highly infectious (crusted scabies). These individuals may not respond to treatment delivered as part of MDA and this might be anticipated to impact on the effectiveness of control programmes. Future modelling studies could be used to explore the impact that crusted scabies cases may have on the efficacy of MDA for scabies control.

Finally, in some settings, epidemics of scabies have been reported to be associated with changing climate conditions, particularly those leading to drought. Modelling could potentially explore whether global climate change is likely to contribute to a rise in the prevalence of scabies or frequency of large-scale outbreaks globally.

## Future work

Control programmes and modelling for scabies are both in their infancy compared to many other NTD programmes. As such, there is considerable scope for modelling to play a valuable role in refining the provisional framework for scabies control and in better estimating the global burden of disease related to scabies.

Modelling approaches will be of value in complementing empirical data in refining several elements of MDA implementation, including better defining the threshold at which MDA should be undertaken for the control of scabies, the level of coverage that must be attained to interrupt transmission, the threshold at which MDA should subsequently be stopped and exploring the optimal number of rounds of MDA to be undertaken to achieve future programmatic goals. Current empirical data on the impact of MDA is predominantly from island populations, which may differ from non-island settings where scabies is a problem. Modelling may help understand differences and additional challenges in conducting MDA in these differing settings.

Secondly, current MDA recommendations include the use of permethrin for individuals unable to receive ivermectin, which results in significant logistical challenges. Age-structured models may allow formal evaluation of different MDA strategies (including choice of drug/s), necessary to justify the additional complexities of the proposed dual-therapy MDA programme. Evaluation of the potential impact of lowering the age of ivermectin administration from five to two years of age can also be explored within these frameworks. These approaches would be challenging to explore in trial situations due to the ethical considerations of not treating young children. Modelling will also have a role to play in predicting the likely impact and potential advantages of moxidectin as an agent for mass control of scabies, compared with currently available ivermectin.

As with other NTD programmes, heterogeneity in small area infection prevalence following MDA is noted within defined intervention zones. Modelling will allow evaluation of the importance of these heterogeneities to rebound of transmission between intervention rounds. In addition, the impact of repeated annual rounds of MDA administration on this heterogeneity will be explored, providing needed evidence to inform programme duration. Current empirical data is based entirely on MDA implementation in rural settings and modelling may help better understand the potential for differential impact of interventions in urban, peri-urban and rural settings.

## Data availability

No data are associated with this article.
