# Supplementary material for: Insights from mathematical modelling on the proposed WHO 2030 goals for scabies
Source: Gates Open Res. 2019 Sep 20;3:1542. [Version 1] doi: 10.12688/gatesopenres.13064.1 (PMC6792349; doi:10.12688/gatesopenres.13064.1)
Supplement: Peer review history

# Peer review: Reviewer response for version 1

**DOI:** 10.21956/gatesopenres.14195.r27914

**Published:** 2019-10-22

**Authors:** Timothy Kinyanjui

**Affiliations:** 1 School of Mathematics, University of Manchester, Manchester, UK

This letter discusses the status of Scabies disease transmission and control and the authors make a case for the use of mathematical modelling in helping inform which are the best control strategies. There are not many rigorous Scabies models and the authors have discussed the only paper in literature that attempts to incorporate mite life stages and drug action in exploring questions related to scabies control. Unlike other diseases, modelling work for Scabies is in its infancy and the authors have discussed potential future work in this area. The letter can be indexed as it is as I think it makes a considerable contribution to the discussion about the control of Scabies.

I confirm that I have read this submission and believe that I have an appropriate level of expertise to confirm that it is of an acceptable scientific standard.

# Peer review: Reviewer response for version 1

**DOI:** 10.21956/gatesopenres.14195.r27912

**Published:** 2019-10-14

**Authors:** Ymkje Stienstra

**Affiliations:** 1 University Medical Center Groningen, Department of Internal Medicine—Infectious Diseases, University of Groningen, Groningen, The Netherlands

This open letter emphasizes the need of MDA for scabies control. The authors discuss the added value of modeling in decision making. Future developments, such as MDA in non-island populations and the use of moxidectin in scabies control, will further increase the role of modeling to optimize MDA strategies. The authors clearly describe these developments. Decision making can be further facilitated by obtaining additional data on scabies complications and socioeconomic impact.

I confirm that I have read this submission and believe that I have an appropriate level of expertise to confirm that it is of an acceptable scientific standard.

# Peer review: Reviewer response for version 1

**DOI:** 10.21956/gatesopenres.14195.r27913

**Published:** 2019-09-26

**Authors:** Jorg Heukelbach

**Affiliations:** 1 Department of Community Health, School of Medicine, Federal University of Ceará, Fortaleza, Brazil

Scabies is a parasitic skin disease, which has been recognized recently by WHO as a Neglected Tropical Disease. The authors of this Open Letter discuss scabies control approaches including mass drug administration, and make reference to mathematical modeling, which has until now been the focus of a single article. The different approaches using topical permethrin and oral ivermectin are discussed, considering available community data. The Letter is publishable as it stands and contributes considerably to the important discussion of scabies control in highly affected communities.

I confirm that I have read this submission and believe that I have an appropriate level of expertise to confirm that it is of an acceptable scientific standard.
